# The value of fibroblast growth factor 21 (FGF21) promoter methylation in the repair and regeneration during the course of chronic hepatitis B

**DOI:** 10.3389/fcell.2025.1643250

**Published:** 2025-09-16

**Authors:** Xue Li, Ying Zhang, Jihui Li, Tong Zhao, YuChen Fan, Shuai Gao, Kai Wang

**Affiliations:** ^1^ Department of Hepatology, Qilu Hospital of Shandong University, Jinan, China; ^2^ Institute of Hepatology, Shandong University, Jinan, China

**Keywords:** FGF21, HBV, liver regeneration, methylation, oxidative stress

## Abstract

**Background:**

Hepatitis B virus (HBV) infection continues to pose a significant threat to global public health. The capacity for liver repair and regeneration plays a critical role in maintaining liver homeostasis during HBV infection. This study investigates the impact of FGF21 promoter methylation on liver repair and regeneration in chronic HBV infection.

**Methods:**

A total of 216 patients with chronic hepatitis B admitted to the Department of Hepatology, Qilu Hospital, Shandong University from October 2023 to October 2024, along with 15 healthy controls, were included in this study. FGF21 promoter methylation levels in peripheral blood mononuclear cells (PBMCs) were assessed using Methlight. Group comparisons were conducted using the Kruskal–Wallis Test, while Spearman correlation analysis was employed to examine the relationship between FGF21 promoter methylation levels and liver injury, repair, and regeneration in chronic HBV patients.

**Results:**

The methylation level of the FGF21 promoter in HBeAg(+) CHB patients was significantly lower compared to HBeAg(−) CHB patients and healthy controls. Additionally, HBeAg(+) CHB patients exhibited significantly higher viral loads and more severe liver damage than HBeAg(−) CHB patients. Spearman correlation analysis revealed that the methylation level of the FGF21 promoter in CHB patients was positively correlated with liver repair and regeneration capacity.

**Conclusion:**

The methylation level of FGF21 serves as an important biomarker for evaluating liver repair and regeneration ability in patients with HBV. It is closely associated with the extent of liver injury and viral load.

## 1 Introduction

The liver plays a crucial role in regulating metabolism, protein synthesis, and detoxification. Following injury, the liver exhibits compensatory adaptation to maintain normal physiological function, with its capacity for repair and regeneration serving as the foundation of its homeostasis. Liver regeneration (LR) is an intricate and vital process that facilitates recovery from liver injury or partial hepatectomy (Michalopoulos and Bhushan, 2021). Hepatocyte proliferation is a key cellular mechanism governing the generation of new hepatocytes during LR, both under stable conditions and following injury (Heinke et al., 2022). Previous studies (Kim et al., 2024) have demonstrated that, after surgical resection of approximately 70% of the liver, the liver can restore total lost liver mass. This restoration occurs through the replication of hepatic epithelial cells, specifically hepatocytes, and biliary epithelial cells: proliferationmediated regeneration. Chronic liver disease (CLD) represents a significant global health challenge. CLD is characterized by progressive hepatic deterioration involving persistent inflammation and parenchymal regeneration, ultimately leading to fibrosis and cirrhosis. The primary etiological factors of CLD include viral hepatitis, chronic alcohol consumption, autoimmune disorders, and genetic conditions. Hepatitis B virus (HBV) infection induces extensive oxidative stress, multiple studies (Jabeen et al., 2021; Mansouri et al., 2018) indicate that HBV alters mitochondrial function, thereby generating oxidative stress and modulating host gene expression. During hepatic injury, necrotic hepatocytes release mitochondrial DNA (mtDNA), which activates the TLR9 and cGAS-STING pathways, promoting neutrophil infiltration and exacerbating liver inflammation (He et al., 2017). Research demonstrates that alterations in mitochondrial metabolism and increased oxidative/nitrosative stress significantly contribute to the pathogenesis and progression of chronic HBV infection (Begriche et al., 2006). Safeguarding hepatocytes from damage and enhancing liver repair and regeneration are pivotal strategies for mitigating liver disease progression (Chalasani et al., 2018). Cytochrome P450 2E1 (CYP2E1) is a major source of reactive oxygen species (ROS) production in the liver (Lu et al., 2017), with its upregulated expression being a critical factor in the development of hepatic oxidative stress (Harjumäki et al., 2021). ROS-induced oxidative stress, mediated by CYP2E1, damages hepatocytes via peroxidation of cellular macromolecules, including lipid peroxidation, protein carbonylation, and DNA oxidation, leading to increased hepatocyte apoptosis and liver fibrosis, thereby impairing the liver’s capacity for repair and regeneration (Zhang et al., 2019). While the role of CYP gene variants in the development and progression of hepatocellular carcinoma (HCC) is well established, limited data exist regarding their correlation with susceptibility to chronic hepatitis B (CHB). Several studies (Bose et al., 2013) have reported that CYP2E1 can significantly influence both the susceptibility and severity of HBV-related liver diseases. Transcriptional coregulators play a crucial role in regulating various physiological processes, including cell survival and death. Among these, nuclear receptor corepressor 1 (NCOR1) serves as an epigenetic regulator of gene transcription (Stallcup and Poulard, 2020). NCOR1 expressed by cardiomyocytes acts as a negative regulator in acute myocardial infarction/reperfusion injury, inhibiting mitochondria-mediated apoptotic pathways and inflammation via the signal transducer and activator of transcription 1 pathway (Qin et al., 2022). Lima et al. (2019) demonstrated that NCOR1 knockdown reduces mitochondrial ROS levels and prevents cell death due to lipid overload in skeletal muscle cells. Another study (Ou-Yang et al., 2018) revealed that hepatocyte-specific NCOR1 deficiency promotes adipogenesis and enhances LR following partial hepatectomy. Inflammation is considered a key factor in coordinating liver injury repair and reconstruction, with leukemia inhibitory factor (LIF), a member of the IL-6 family of inflammatory cytokines (Xie et al., 2025), playing a significant role. LIF signals through its receptor LIR (LIFR) and co-receptor gp130 to activate the JAK/STAT inflammatory pathway (Guo et al., 2021). Yao et al. (2021) demonstrated that LIFR expression was significantly downregulated in hepatocellular carcinoma tissues, and the absence of LIFR facilitated the progression of liver cancer. Deng et al. (2024) reported that LIFR promoted liver injury repair and regeneration by enhancing neutrophil recruitment and secreting hepatocyte growth factor, thereby accelerating hepatocyte proliferation and regeneration. Studies (Li et al., 2023; Frick et al., 2024) have indicated that platelet (PLT) not only play a crucial role in physiological hemostasis but also contribute significantly to liver ischemia-reperfusion injury, liver injury, tissue repair, and LR. A reduced PLT count can lead to spontaneous bleeding, infections, and other complications, which can severely impact patient prognosis. Moreover, both platelet count and serotonin levels in platelets are associated with post-hepatectomy liver dysfunction and morbidity (Amygdalos et al., 2020). Collectively, these findings underscore the critical role of PLT in promoting new cell production and LR.

Fibroblast growth factor21 (FGF21) is an atypical member of the fibroblast growth factor family, exhibiting distinct activities in cell proliferation, angiogenesis, oxidative stress response, and tissue repair and regeneration (Itoh et al., 2016). FGF21 expression is upregulated in response to both physiological and pathophysiological stress. In particular, under pathological conditions such as metabolic syndrome, FGF21 levels are compensatorily increased in response to oxidative stress, endoplasmic reticulum stress, and mitochondrial dysfunction (Li et al., 2024). Recent studies have demonstrated that FGF21 exerts a protective effect on the liver following acute insults that induce damage (Ma et al., 2023; Eguchi et al., 2020). For instance, Huai et al. (2024) reported that overexpression of FGF21 in mesenchymal stem cells/stromal cells significantly enhanced therapeutic efficacy in mice with alcohol-related liver disease, likely by mitigating liver damage, steatosis, inflammatory infiltration, oxidative stress, hepatocyte apoptosis, and promoting liver regeneration. Mechanistically, FGF21 enhances the immunomodulatory function of mesenchymal stem cells on macrophages. Currently, FGF21 and hepatocyte growth factor receptor have emerged as promising therapeutic targets in LR (Falamarzi et al., 2022). Therefore, FGF21 may serve as a sensitive, specific, and clinically significant biomarker with predictive value for liver function assessment and the progression and prognosis of liver disease. In this study, we primarily utilized MethyLight technology to evaluate the methylation levels of the FGF21 promoter in patients with CHB and healthy controls (HC). Additionally, we investigated the expression levels of FGF21, LIFR, and CYP2E1 to elucidate the potential relationship between FGF21 promoter methylation and the expression of molecules involved in hepatic repair and regeneration in CHB patients.

## 2 Materials and methods

### 2.1 Participants

**FIGURE 1:**
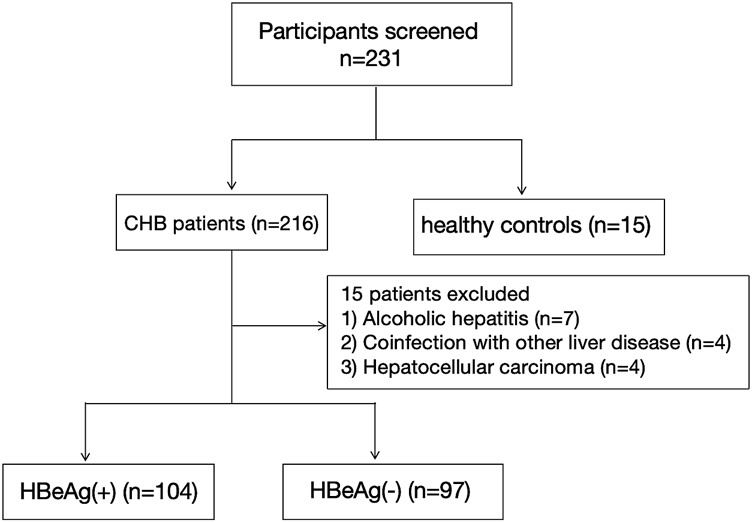
Flowchart for the enrollment of participants.

The subjects were recruited from the Department of Hepatology at Qilu Hospital of Shandong University between October 2023 and October 2024. The Medical Ethical Committee of Qilu Hospital of Shandong University approved this study, with the ethical approval number “KYLL-202306–021-1”. Informed consent was obtained from all individual participants included in the study. All procedures of this study were in accordance with the Declaration of Helsinki. The participant selection process is illustrated in Figure 1. The inclusion criteria were as follows: (1) aged 18 years or older; (2) positive serum hepatitis B surface antigen (HBsAg) for a minimum duration of 6 months; (3) all enrolled patients met the criteria specified in the 2015 Asia Pacific Association for the Study of the Liver (APASL) Practice Guidelines for the Management of CHB (Sarin et al., 2016). Exclusion criteria included: (1) presence of autoimmune or metabolic liver disease, infection with hepatitis viruses other than HBV or human immunodeficiency virus (HIV), drug-induced hepatitis, or alcoholic hepatitis; (2) pregnancy; (3) HCC.

### 2.2 Observation indicators

The observation indicators included in this study included age, gender, serum biochemical parameters (ALT, AST, TBIL, ALB, AFP and PLT), and HBV serological parameters (including HBsAg, HBeAg, and HBV-DNA). In addition, we measured the FGF21, LIFR, NCOR1 and CYP2E1 mRNA level, the FGF21 promoter methylation level and the plasma FGF21 level.

### 2.3 DNA extraction and sodium bisulfite modification

According to the specified protocol, peripheral blood mononuclear cells (PBMCs) were isolated via density gradient centrifugation using Ficoll-Paque (Pharmacia Diagnostics, Uppsala, Sweden) and subsequently stored at −80 °C. Genomic DNA was then extracted from the PBMCs following the standard operating procedure outlined in the QIAamp DNA Blood Mini Kit (QIAGEN, Valencia, CA, United States). The extracted DNA underwent sodium bisulfite conversion using the EZ DNA Methylation-Gold kit (Zymo Research, Orange, CA, United States), strictly adhering to the manufacturer’s instructions. The modified DNA samples were preserved at −80 °C until further analysis.

### 2.4 RNA extraction and RT-qPCR

In this study, total RNA was extracted from PBMCs using TRIzol Reagent (Invitrogen). Subsequently, cDNA synthesis was performed according to the protocol provided by the RevertAid First Strand cDNA Synthesis Kit (Thermo Fisher Scientific, formerly Fermentas, Vilnius, Lithuania). The synthesized cDNA was used as a template for reverse-transcriptase quantitative polymerase chain reaction (RT-qPCR), which was conducted on the CFX Connect real-time PCR system (Bio-Rad Laboratories, Hercules, CA) for real-time detection.

### 2.5 TaqMan probe-based quantitative methylation-specific polymerase chain reaction (MethyLight)

The MethyLight method was employed to assess the methylation levels of the FGF21 gene promoter region in all subjects. Specific primers and probes for the FGF21, LIFR, NCOR1, CYP2E1, and B-actin genes are detailed in Table 1. The methylation status of the FGF21 promoter was quantified as a Percentage of Methylated Reference (PMR). PMR = 100% x 2^-[Δ^Ct (target gene-control gene) Sample- ΔCt (target gene-control gene) Reference] (Gao et al., 2015).

**TABLE 1:** Sequences of used primers and probes.

Gene	Forward primer sequence (5′-3′)	Primer/probe sequence (5′-3′)
RT-qPCR		
FGF21	CTGCAGCTGAAAGCCTTGAAGC	GTATCCGTCCTCAAGAAGCAGC
LIFR	CACCTTCCAAAATAGCGAGTATGG	ATGGTTCCGACCGAGACGAGTT
NCOR1	AGACAGCAGTCCTGAGAAAGGC	GCTGTTCTTGGACTCCTAGTCC
CYP2E1	GAGCACCATCAATCTCTGGACC	CACGGTGATACCGTCCATTGTG
ACTB	ATGGGTCAGAAGGATTCCTATGTG	CTTCATGAGGTAGTCAGTCAGGTC
Methylight		
FGF21	TTATTAAGACGTAGAGATCGGTAGT	TCACGTAACTTACTTAACCTTATCAAT
ACTB	TGGTGATGGAGGAGGTTTAGTAAGT	AACCAATAAAACCTACTCCTCCCTTAAA
Probe oligo sequence		
FGF21	AACGACTCACCCTCCTTATCCTACCC	
ACTB	ACCACCACCCAACACACAATAACAAACACA	

### 2.6 Enzyme-linked immunosorbent assay (ELISA)

Plasma FGF21 concentrations were measured using an ELISA assay. The assays were performed by Lengton Bioscience Co, Shanghai, China, employing a competitive method for sample content detection. Absorbance was measured at 450 nm following the manufacturer’s protocol.

### 2.7 Statistical analysis

Quantitative variables were expressed as median (cen-tile 25; centile 75). Categorical variables were expressed as number (percentage). The data were analyzed using SPSS 27.0 statistical software (SPSS Inc., Chicago, IL, United States) and GraphPad Prism 9.0 (San Diego, CA, United States). The Kruskal–Wallis H test was employed to evaluate intergroup differences in continuous variables. Spearman’s rank correlation coefficient was utilized to investigate the association between the methylation status of the FGF21 promoter and factors related to repair and regeneration. A *P* value of less than 0.05 was considered to indicate statistical significance.

## 3 Results

### 3.1 General characteristics

The process for study selection and exclusion is illustrated in Figure 1. Initially, a total of 231 participants were included, consisting of 216 patients with chronic hepatitis B and 15 healthy controls. After excluding 15 patients who did not meet the inclusion criteria, a total of 216 participants were finally included in the study, among which 104 were HBeAg (+), 97 were HBeAg (−), and 15 were HCs. The basic characteristics of these groups are presented in Table 2.

**TABLE 2:** Baseline characteristics of the individuals enrolled in the study.

Parameter	HCs(15)	HBeAg(−) (97)	HBeAg(+) (104)	*P* value
Age (years)	34.00 (28.00,50.00)	41.00 (34.50,48.50)	39.00 (35.00,47.00)	0.397^b^
Male, n (%)	4 (26.67)	63 (64.95)	69 (66.35)	<0.001^c^
HBsAg(IU/mL)	NA	2,259.24 (550.42,5525.86)	3,077.50 (1,279.87,7857.85)	0.524^a^
HBeAg(IU/mL)	NA	0.42 (0.37,0.63)	44.20 (2.70,852.72)	<0.001^a^
log10 [HBV-DNA]	NA	3.55 (3.03,4.28)	3.86 (3.20,4.98)	0.206^a^
AFP(ng/mL)	3.08 (2.65,3.11)	2.53 (1.87,3.54)	2.78 (2.04,3.75)	0.342^b^
ALT (U/L)	15.00 (10.00,19.00)	47.00 (44.00,52.00)	50.0 (45.25,59)	<0.001^b^
AST (U/L)	18.00 (14.50,19.50)	39.00 (35.00,45.00)	44.50 (36.00,52.75)	<0.001^b^
TBil (umol/L)	10.40 (7.30,11.90)	11.70 (8.50,15.30)	11.30 (8.15,16.27)	0.351^b^
Alb(g/L)	48.90 (45.55, 50.45)	48.15 (46.05,50.00)	47.90 (46.03,50.30)	0.856^b^
PLT	255.00 (227.00,324.00)	212.00 (178.50,216.00)	199.00 (151.25,224.75)	<0.001^b^
CYP2E1	0.17 (0.10,0.18)	0.10 (0.07,0.17)	0.15 (0.11,0.19)	0.970^b^
NCOR1	0.07 (0.06,0.10)	0.10 (0.06,0.18)	0.12 (0.87,0.17)	<0.001^b^
LIFR	0.16 (0.11,0.37)	0.16 (0.10,0.28)	0.07 (0.04,0.12)	<0.001^b^
mRNA	2.02 (0.14,9.10)	3.88 (2.09,11.87)	23.89 (5.91,46.14)	<0.001^b^
PMR (%)	17.30 (16.40,20.24)	16.37 (14.27,18.16)	15.00 (13.74,16.55)	<0.001^b^
FGF21 (ng/mL)	973.37 (602.66,1204.02)	938.97 (643.39,1214.90)	1,189.39 (870.62,1693.14)	<0.001^b^
8-OHdG (ng/mL)	1.15 (0.68,4.13)	5.82 (3.14,10.16)	10.09 (5.54,16.49)	<0.001^b^
CAT(ng/mL)	27.00 (25.00,35.68)	20.07 (13.81,28.00)	17.98 (15.60,20.32)	0.009^b^
SOD (ng/mL)	12.75 (10.74,13.92)	10.35 (8.35,12.49)	8.86 (7.34,10.14)	0.001^b^

Quantitative variables were expressed as medians (25th, 75th).

Qualitative variables were expressed as number (percentage).

^a^ Mann–Whitney U test. ^b^Kruskal–Wallis H test. ^c^Chi-square test.

### 3.2 Expression of FGF21 in chronic hepatitis B with different HBeAg serologic status

**FIGURE 2:**
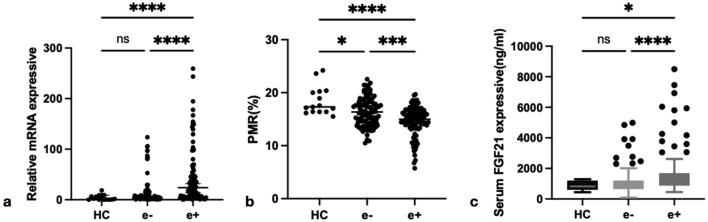
The expression of FGF21 in different groups. **(a)** Relative mRNA level of FGF21 in PBMCs from HCs, HBeAg(−) and HBeAg(+). Relative mRNA level of FGF21 was significantly higher in HBeAg(+) than in HBeAg(+) (*P* < 0.0001) and HCs(*P* < 0.0001), by using the Kruskal–Wallis Test and Dunn’s test. **(b)** FGF21 promoter methylation level in HBeAg (+) group was significantly lower than that in HBeAg (−) group (*P* < 0.001) and HC group (*P* < 0.0001), respectively,by using the Kruskal–Wallis Test and Dunn’s test. **(c)** Serum FGF21 level from HCs, HBeAg(−) and HBeAg(+). Serum FGF21 level was significantly higher in HBeAg(+) than in HBeAg(−) (*P* < 0.0001) and HCs(*P* < 0.05),respectively, by using the Kruskal–Wallis Test and Dunn’s test. ns, *P* > 0.05; *, *P* ≤ 0.05; **, *P* ≤ 0.01; ***, *P* ≤ 0.001; ****, *P* ≤ 0.0001.

To elucidate the significance of FGF21 in CHB, we conducted a comprehensive analysis of FGF21 expression levels across different HBeAg serological statuses. Specifically, we quantified the relative mRNA levels of FGF21 in PBMCs from HBeAg(+), HBeAg(−) CHB patients, and HCs (Figure 2a). The results demonstrated that the relative expression level of FGF21 mRNA was significantly elevated in CHB patients compared to HCs (P < 0.0001), with a further significant increase observed in the HBeAg(+) group relative to the HBeAg(−) group (*P* < 0.0001). Subsequently, we evaluated the promoter methylation levels of FGF21 in the same patient groups, expressed as percent promoter methylation ratio (PMR) (Figure 2b). Figure 2a Our findings revealed that the promoter methylation level of FGF21 was markedly lower in the HBeAg(+) group compared to both the HBeAg(−) group (*P* < 0.001) and HCs (*P* < 0.0001). Additionally, we assessed serum FGF21 levels and found them to be significantly higher in the HBeAg(+) group compared to the HBeAg(−) group (*P* < 0.0001) and HCs (*P* < 0.05) (Figure 2c).

**FIGURE 3:**
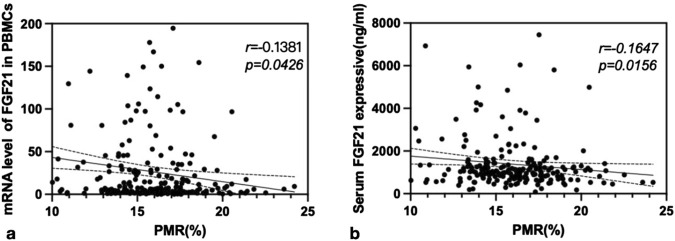
The associations between FGF21 promoter methylation level and mRNA level in PBMCs, and FGF21 expressive in serum. Significant correlation was observed between the PMR value of FGF21 and mRNA level in PBMCs **(a)** Spearman’s *r* = −0.1381, *P* = 0.0426), and FGF21 expressive in serum **(b)** Spearman’s r = −0.1647, P = 0.0156).

Spearman rank correlation analysis was conducted to investigate the relationship between FGF21 methylation levels and both FGF21 mRNA levels in PBMCs and serum FGF21 expression levels in patients with CHB. The results demonstrated that the PMR value of FGF21 showed a significant negative correlation with FGF21 mRNA levels in PBMCs (Spearman’s *r* = −0.1381, *P* = 0.0426) and serum FGF21 expression levels (Spearman’s *r* = −0.1647, *P* = 0.0156), as shown in Figure 3.

### 3.3 The expression levels of repair and regeneration factors varied among patients with different HBeAg serologic statuses in both the HC and CHB groups

**FIGURE 4:**
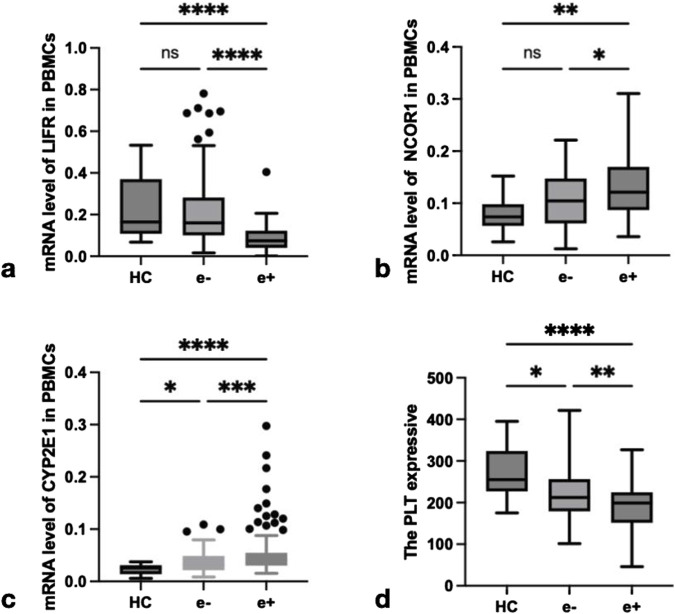
The expression of LIFR, NCOR1,CYP2E1 and PLT in different groups. **(a)** Relative mRNA level of LIFR in PBMCs from HCs, HBeAg(−) and HBeAg(+). Relative mRNA level of LIFR was significantly lower in HBeAg(+) than in HBeAg(−) (*P* < 0.0001) and HCs(*P* < 0.0001), respectively, by using the Kruskal–Wallis Test and Dunn’s test. **(b)** Relative mRNA level of NCOR1 in PBMCs from HCs, HBeAg(−) and HBeAg(+). Relative mRNA level of NCOR1 was significantly higher in HBeAg(+) than in HBeAg(−) (*P* < 0.05) and HCs(*P* < 0.01), respectively, by using the Kruskal–Wallis Test and Dunn’s test. **(c)** Relative mRNA level of CYP2E1 in PBMCs from HCs, HBeAg(−) and HBeAg(+). Relative mRNA level of CYP2E1 was significantly higher in HBeAg(+) than in HBeAg(−) (*P* < 0.001) and HCs(*P* < 0.0001),respectively, by using the Kruskal–Wallis Test and Dunn’s test. **(d)** The level of PLT from HCs, HBeAg(−) and HBeAg(+). The PLT level was significantly lower in HBeAg(+) than in HBeAg(−) (*P* < 0.01) and HCs(*P* < 0.0001),respectively, by using the Kruskal–Wallis Test and Dunn’s test. ns, *P* > 0.05; *, *P* ≤ 0.05; **, *P* ≤ 0.01; ***, *P* ≤ 0.001; ****, *P* ≤ 0.0001.

To investigate the roles of repair, regeneration, and damage factors LIFR, NCOR1, CYP2E1, and PLT in CHB and their relationship with FGF21, this study examined the expression levels of LIFR, NCOR1, and CYP2E1 mRNA in PBMCs from CHB patients with different HBeAg serologic statuses. The expression levels of LIFR, NCOR1, CYP2E1, and platelet counts were compared across groups. As illustrated in Figure 4, the relative mRNA levels of LIFR in HBeAg(+) patients were significantly lower than those in HBeAg(−) patients (*P* < 0.0001) and HCs (*P* < 0.0001) (Figure 4a). The relative mRNA level of NCOR1 was significantly higher in HBeAg(+) patients compared to HBeAg(−) patients (*P* < 0.05) and HCs (*P* < 0.01) (Figure 4b). The relative mRNA level of CYP2E1 was significantly elevated in HBeAg(+) patients compared to HBeAg(−) patients (*P* < 0.001) and HCs (*P* < 0.0001) (Figure 4c). PLT counts were significantly lower in HBeAg(+) patients compared to HBeAg(−) patients (*P* < 0.01) and HCs (*P* < 0.0001) (Figure 4d).

### 3.4 Analysis of the correlation between repair and regeneration factors and HBV viral load

**FIGURE 5:**
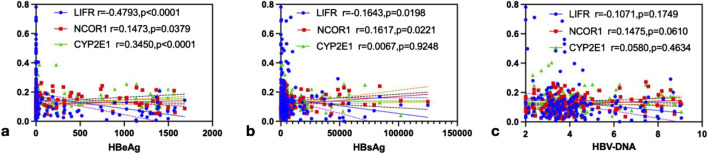
The correlation analysis of LIFR, NCOR1 and CYP2E1 with HBeAg, HBsAg and HBV-DNA. **(a)** HBeAg was negatively correlated with LIFR (*r* = −0.4793, *P* < 0.0001), and positively correlated with NCOR1 (*r* = 0.1473, *P* = 0.0379) and CYP2E1 (*r* = 0.3450, *P* < 0.0001),respectively, by Spearman’s correlation analysis. **(b)** HBsAg was negatively correlated with LIFR (*r* = −0.1643, *P* = 0.0198), and positively correlated with NCOR1 (*r* = 0.1617, *P* = 0.0221) and CYP2E1 (*r* = 0.0067, *P* = 0.9248),respectively, by Spearman’s correlation analysis. **(c)** HBV-DNA was negatively correlated with LIFR (*r* = −0.1071, *P* = 0.1749), and positively correlated with NCOR1 (*r* = 0.1475, *P* = 0.0610) and CYP2E1 (*r* = 0.0580, *P* = 0.4634),respectively, by Spearman’s correlation analysis.

Spearman rank correlation analysis was conducted to investigate the associations between viral markers (HBeAg, HBsAg, and HBV-DNA) and mRNA levels of LIFR, NCOR1, and CYP2E1 in PBMCs from CHB patients. The results revealed that HBeAg exhibited a significant negative correlation with LIFR (*r* = −0.4793, *P* < 0.0001), a positive correlation with NCOR1 (*r* = 0.1473, *P* = 0.0379), and a positive correlation with CYP2E1 (*r* = 0.3450, *P* < 0.0001) (Figure 5a). HBsAg showed a significant negative correlation with LIFR (*r* = −0.1643, *P* = 0.0198), a positive correlation with NCOR1 (*r* = 0.1617, *P* = 0.0221), and no significant correlation with CYP2E1 (*r* = 0.0067, *P* = 0.9248) (Figure 5b). HBV-DNA demonstrated a non-significant negative correlation with LIFR (*r* = −0.1071, *P* = 0.1749), a non-significant positive correlation with NCOR1 (*r* = 0.1475, *P* = 0.0610), and a non-significant positive correlation with CYP2E1 (*r* = 0.0580, *P* = 0.4634) (Figure 5c).

### 3.5 The association between promoter methylation levels of the FGF21 gene and expression of repair and regeneration factors in relation to liver function in patients with chronic hepatitis B

**FIGURE 6:**
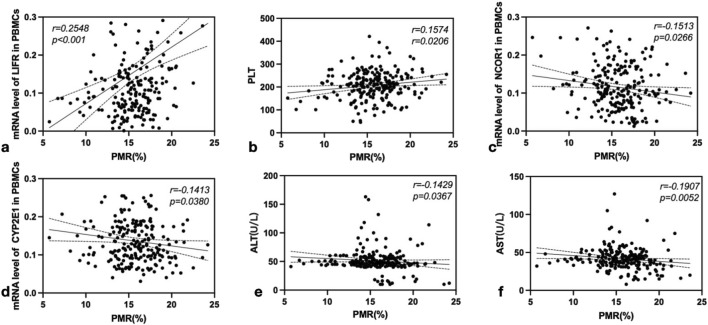
Correlation analysis of the PMR value of FGF21 with LIFR, PLT, NCOR1, CYP2E1, ALT and AST. **(a)** The PMR value of FGF21 was positively correlated with the expression of LIFR mRNA (*r* = 0.2548, *P* < 0.001). **(b)** The PMR value of FGF21 was positively correlated with the PLT level (*r* = 0.1574, *P* = 0.0206). **(c)** The PMR value of FGF21 was negatively correlated with the expression of NCOR1 mRNA (*r* = -0.1513, *P* = 0.0266). **(d)** The PMR value of FGF21 was negatively correlated with the expression of CYP2E1 mRNA (*r* = −0.1413, *P* = 0.0380). **(e)** The PMR value of FGF21 was negatively correlated with the ALT level (*r* = −0.1429, *P* = 0.0367). **(f)** The PMR value of FGF21 was negatively correlated with the AST level (*r* = −0.1907, *P* = 0.0052).

Spearman rank correlation analysis was conducted to further investigate the relationships between FGF21 promoter methylation levels, expression levels of repair and regeneration factors, and liver function. The results are presented in Figure 6. As illustrated in the figure, the PMR value of FGF21 showed a significant positive correlation with LIFR mRNA expression (*r* = 0.2548, *P* < 0.001, Figure 6a) and PLT levels (*r* = 0.1574, *P* = 0.0206, Figure 6b). Conversely, the FGF21 PMR value exhibited significant negative correlations with NCOR1 mRNA expression (*r* = −0.1513, *P* = 0.0266, Figure 6c), CYP2E1 mRNA expression (*r* = −0.1413, *P* = 0.0380, Figure 6d), ALT levels (*r* = −0.1429, *P* = 0.0367, Figure 6e), and AST levels (*r* = −0.1907, *P* = 0.0052, Figure 6f).

### 3.6 The relationship between promoter methylation of the FGF21 gene and viral load in patients with chronic hepatitis B

As illustrated in Table 3, the Spearman rank correlation test revealed that the promoter methylation level of FGF21 was significantly negatively correlated with HBV-DNA (*r* = −0.2460, *P* = 0.0016) and HBsAg (*r* = −0.1697, *P* = 0.0016). Furthermore, it exhibited a significant negative correlation with HBeAg (*r* = −0.1749, *P* = 0.0132).

**TABLE 3:** Correlation analysis between PMR and viral load.

Parameter	PMR (%)	
	*r* value	*P* Value
HBV-DNA	−0.2460	0.0016
HBsAg	−0.1697	0.0160
HBeAg	−0.1749	0.0132

## 4 Discussion

The liver plays a crucial role in various physiological processes, including metabolism, immune defense, and detoxification. HBV infection represents a significant global public health challenge, affecting approximately 2 billion individuals worldwide, its associated liver cirrhosis and HCC (GBD, 2015 LRI Collaborators, 2017). FGF21 is a stress-responsive factor produced by the liver, whose expression and induction are believed to confer protective effects when hepatic homeostasis is disrupted by diverse stimuli (Desai et al., 2017; Ye et al., 2014). Yang et al. (2013) demonstrated that overexpression of FGF21 in mice promotes LR following partial hepatectomy. Qiang et al. (2021) revealed that FGF21 plays a critical role in hepatocyte survival, and exogenous administration of FGF21 enhances liver resilience by activating autophagy, reducing oxidative stress, and inhibiting apoptosis. These findings confirm that FGF21 activates hepatocyte autophagy via the AMPK-mTOR signaling pathway, thereby accelerating the regeneration of damaged liver cells. In a mouse model of nonalcoholic steatohepatitis using leptin-deficient mice fed a methionine and choline-deficient diet, an FGF21 analogue was shown to reverse liver inflammation and fibrosis (Stefan et al., 2023; Keinicke et al., 2020). However, Liu et al. (2022) reported that elevated FGF21 levels are associated with poorer survival rates in HCC patients, suggesting that increased serum FGF21 may serve as a prognostic indicator for HCC. While FGF21 enhances liver regeneration by inhibiting apoptosis and reducing oxidative stress, thereby improving the survival of damaged hepatocytes (Qiang et al., 2021), it has been proposed (Gómez-Sámano et al., 2017) that the initial increase in FGF21 production during the early stages of disease represents a compensatory response by the organism. Conversely, other researchers suggest that sustained elevation of serum FGF21 as the disease progresses may lead to the development of resistance to FGF21. In peripheral nerve injury (PNI), studies (Lu et al., 2019) have demonstrated that FGF21 exerts protective effects on bone marrow and nerve regeneration following PNI, potentially through inhibition of the overactivation of the ERK/Nrf-2 signaling pathway. The liver demonstrates a remarkable capacity for regeneration in response to injury or viral infection. Multiple growth factors and cytokines are intricately involved in regulating this regenerative process. The liver’s regenerative ability is essential for maintaining homeostasis; however, this capacity is markedly diminished in cases of severe or CLD (Huang et al., 2022). When viral activation results in extensive hepatocyte death and the residual functional liver mass becomes insufficient, the liver’s regenerative potential may be compromised, potentially leading to liver failure (Clavien et al., 2007). Walesky et al. (2020) demonstrated that cytokines, growth factors, compensatory mechanisms, and epigenetic factors play pivotal roles in LR. Epigenetic regulators not only influence cell proliferation and stem cell differentiation but also significantly contribute to the severity of liver injury and the maintenance of tissue microenvironment homeostasis. DNA methylation, which involves the addition of methyl groups to DNA, represents one of the most significant epigenetic modifications. Previous studies have demonstrated that DNA methylation is intricately associated with the development of cancer, oxidative stress, and validation. Given the pressing concern regarding the impact of HBV infection on liver regeneration, our study investigates the effect of FGF21 promoter methylation levels on liver function assessment and repair-regeneration capacity in the context of HBV infection.

Currently, biomarkers based on PBMCs have great potential for clinical application. Numerous studies have utilized them for early diagnosis or efficacy monitoring of diseases. Previous studies (Feng et al., 2015) have demonstrated a positive correlation between peripheral blood and liver tissue characteristics in patients with chronic HBV infection and both ALT levels and the extent of liver damage. Chen et al. (2019) utilized animal models to show that the mRNA and protein expression levels of BATF in liver tissues of HBV transgenic mice, as well as the serum concentrations of Th17 and the cytokines IL-17 and IL-22, were significantly elevated compared to those in the control group. In the author’s prior research, it was also observed that the proportion of Th17 in PBMCs was markedly higher in patients with chronic HBV infection. Fan et al. (2016) reported that the relative expression level of A20 mRNA in PBMCs from patients with HCC was significantly higher than that in CHB, and elevated A20 protein expression was similarly detected in HCC liver tissues. In the study of HBV-ACLF disease, Li et al. (2022) conducted functional synergy analysis of seven biological processes related to PBMCs responses and the top 500 differentially expressed genes (DEGs), showing that the viral process was associated with all disease stages. These findings collectively support the significant clinical translational potential of PBMCs-based biomarkers, which can be applied for disease early warning, staging, and therapeutic monitoring. In this study, we mainly used PBMCs for the research, and subsequently we utilized quantitative RT-PCR, MethLight, and ELISA to evaluate FGF21 expression and elucidate the relationship between FGF21 promoter methylation levels and liver repair-regeneration in CHB patients. Our findings revealed that PBMCs and serum FGF21 expression levels were elevated in CHB patients compared to HCs, while methylation levels were reduced. Specifically, HBeAg(+) CHB patients exhibited higher PBMCs and serum FGF21 expression levels and lower methylation levels than HBeAg(−) patients. These results suggest that the increased FGF21 expression in PBMCs of CHB patients is a compensatory response to HBV viral activation and oxidative stress-induced damage. Additionally, LIFR mRNA expression and PLT levels in PBMCs of HBeAg(+) patients were significantly lower than those in HBeAg(−) patients and healthy controls. Similarly, NCOR1 and CYP2E1 mRNA levels, as well as ALT and AST levels, were also lower in HBeAg(+) patients compared to HBeAg(−) patients. These observations indicate that LIFR and PLT play crucial roles in the repair and regeneration processes during the progression of CHB. In the correlation analysis between viral load and repair and regeneration factors, LIFR mRNA exhibited a significant negative correlation with HBeAg, while NCOR1 and CYP2E1 mRNA showed significant positive correlations with HBeAg. These findings suggest that liver repair and regeneration capacity is associated with HBeAg status. Our previous studies have demonstrated that HBeAg status reflects the body’s response to oxidative stress, which may be linked to virus activation and liver damage in the progression of CHB. Further analysis revealed that HBsAg levels were negatively correlated with LIFR mRNA and positively correlated with NCOR1 mRNA, indicating that higher viral loads impair liver repair and regeneration. We hypothesize that the expression of repair and regeneration factors in CHB patients may be influenced by the methylation status of the FGF21 promoter.

Therefore, the correlation analysis between the methylation level of the FGF21 promoter and the mRNA expression levels of LIFR, NCOR1, and CYP2E1, as well as PLT, ALT, and AST, revealed that the methylation level of FGF21 was significantly positively correlated with LIFR mRNA expression and PBMC PLT levels. Conversely, it exhibited significant negative correlations with NCOR1 and CYP2E1 mRNA expression, and ALT and AST levels, supporting our hypothesis. Additionally, we performed a correlation analysis between the methylation level of the FGF21 promoter and viral load. The results indicated a significant negative correlation between the methylation level of the FGF21 promoter and HBV-DNA, HBsAg, and HBeAg levels, further validating our conclusion that the methylation status of the FGF21 promoter is closely associated with the progression and development of CHB. This association is linked to liver injury induced by viral activation and oxidative stress, and plays a crucial role in liver repair and regeneration following injury, potentially serving as an important biomarker for these processes. In summary, this study underscores the regenerative potential of FGF21 in the context of HBV infection, offering promising prospects for the diagnosis and treatment of CHB. Biomarkers indicative of liver regenerative capacity may enhance patient outcomes in CHB management. However, our research has certain limitations. Firstly, we evaluated the methylation level of the FGF21 promoter in PBMCs, but the methylation situation in the liver is still unknown. In the future, we will examine the methylation status and gene expression in liver tissues. Secondly, future research should adopt a large-scale, multi-center approach to verify the accuracy of these findings, and conduct mechanism studies by integrating public databases and *in vitro*/*in vivo* experiments to elucidate how methylation of the FGF21 promoter affects its expression and signal transduction, and subsequently influences liver repair and regeneration functions.

## 5 Conclusion

The methylation status of FGF21 serves as a critical indicator for evaluating the liver’s repair and regeneration capacity in patients with CHB. This marker is closely associated with both the extent of hepatic injury and viral load.

## Data Availability

The original contributions presented in the study are included in the article/supplementary material, further inquiries can be directed to the corresponding authors.

## Abbreviations

HBV, Hepatitis B virus; FGF21, Fibroblast growth factor 21; LIF, Leukemia inhibitory factor; LIFR, LIF receptor; NCOR1, Nuclear receptor corepressor 1; CYP2E1, Cytochrome P450 2E1; LR, Liver regeneration; HBeAg, Hepatitis B e Antigen; HBsAg, Hepatitis B surface antigen; CLD, Chronic liver disease; CHB, Chronic hepatitis B; HCs, Healthy controls; PMR, The percentage of methylation reference; PBMCs, Peripheral blood mononuclear cells; ROS, Reactive oxygen species; HCC, Hepatocellular carcinoma; ALT, Alanine aminotransferase; AST, Aspartate aminotransferase; TBIL, Total bilirubin; ALB, Albumin; AFP, Alpha-fetoprotein; PLT, platelet

## References

[B1] Amygdalos I. Czigany Z. Bednarsch J. Boecker J. Santana D. A. M. Meister F. A. (2020). Low postoperative platelet counts are associated with major morbidity and inferior survival in adult recipients of orthotopic liver transplantation. J. Gastrointest. Surg. 24 (9), 1996–2007. 10.1007/s11605-019-04337-3 31388889

[B2] Begriche K. Igoudjil A. Pessayre D. Fromenty B. (2006). Mitochondrial dysfunction in NASH: causes, consequences and possible means to prevent it. Mitochondrion 6 (1), 1–28. 10.1016/j.mito.2005.10.004 16406828

[B3] Bose S. Tripathi D. M. Sukriti S. Sakhuja P. Kazim S. N. Sarin S. K. (2013). Genetic polymorphisms of CYP2E1 and DNA repair genes HOGG1 and XRCC1: association with hepatitis B related advanced liver disease and cancer. Gene 519 (2), 231–237. 10.1016/j.gene.2013.02.025 23454624

[B4] Chalasani N. Younossi Z. Lavine J. E. Charlton M. Cusi K. Rinella M. (2018). The diagnosis and management of nonalcoholic fatty liver disease: practice guidance from the American association for the study of liver diseases. Hepatology 67 (1), 328–357. 10.1002/hep.29367 28714183

[B5] Chen L. Y. Fan X. P. Fan Y. C. Zhao J. Gao S. Li F. (2019). BATF interference blocks Th17 cell differentiation and inflammatory response in hepatitis B virus transgenic mice. Dig. Dis. Sci. 64 (3), 773–780. 10.1007/s10620-018-5392-x 30498928

[B6] Clavien P. A. Petrowsky H. DeOliveira M. L. Graf R. (2007). Strategies for safer liver surgery and partial liver transplantation. N. Engl. J. Med. 356 (15), 1545–1559. 10.1056/NEJMra065156 17429086

[B7] Deng Y. Zhao Z. Sheldon M. Zhao Y. Teng H. Martinez C. (2024). LIFR regulates cholesterol-driven bidirectional hepatocyte-neutrophil cross-talk to promote liver regeneration. Nat. Metab. 6 (9), 1756–1774. 10.1038/s42255-024-01110-y 39147934 PMC11498095

[B8] Desai B. N. Singhal G. Watanabe M. Stevanovic D. Lundasen T. Fisher F. M. (2017). Fibroblast growth factor 21 (FGF21) is robustly induced by ethanol and has a protective role in ethanol associated liver injury. Mol. Metab. 6 (11), 1395–1406. 10.1016/j.molmet.2017.08.004 29107287 PMC5681240

[B9] Eguchi Y. Wong G. Lee E. I. Akhtar O. Lopes R. Sumida Y. (2020). Epidemiology of non-alcoholic fatty liver disease and non-alcoholic steatohepatitis in Japan: a focused literature review. JGH Open 4 (5), 808–817. 10.1002/jgh3.12349 33102749 PMC7578337

[B10] Falamarzi K. Malekpour M. Tafti M. F. Azarpira N. Behboodi M. Zarei M. (2022). The role of FGF21 and its analogs on liver associated diseases. Front. Med. (Lausanne) 9, 967375. 10.3389/fmed.2022.967375 36457562 PMC9705724

[B11] Fan Y. C. Zhang Y. Y. Sun Y. Y. Wang N. Xiao X. Y. Wang K. (2016). Altered expression of A20 gene in peripheral blood mononuclear cells is associated with the progression of chronic hepatitis B virus infection. Oncotarget 7 (42), 68821–68832. 10.18632/oncotarget.11993 27634895 PMC5356592

[B12] Feng H. Yin J. Han Y. P. Zhou X. Y. Chen S. Yang L. (2015). Regulatory T cells and IL-17(+) T helper cells enhanced in patients with chronic hepatitis B virus infection. Int. J. Clin. Exp. Med. 8 (6), 8674–8685. 26309519 PMC4538083

[B13] Frick J. Frobert A. Quintela Pousa A. M. Balaphas A. Meyer J. Schäfer K. (2024). Evidence for platelet-derived transforming growth factor β1 as an early inducer of liver regeneration after hepatectomy in mice. FASEB J. 38 (17), e70039. 10.1096/fj.202400345R 39258958

[B14] Gao S. Sun F. K. Fan Y. C. Shi C. H. Zhang Z. H. Wang L. Y. (2015). Aberrant GSTP1 promoter methylation predicts short-term prognosis in acute-on-chronic hepatitis B liver failure. Aliment. Pharmacol. Ther. 42 (3), 319–329. 10.1111/apt.13271 26040771

[B15] GBD 2015 LRI Collaborators (2017). Estimates of the global, regional, and national morbidity, mortality, and aetiologies of lower respiratory tract infections in 195 countries: a systematic analysis for the global burden of disease study 2015. Lancet Infect. Dis. 17 (11), 1133–1161. 10.1016/S1473-3099(17)30396-1 28843578 PMC5666185

[B16] Gómez-Sámano M. Á. Grajales-Gómez M. Zuarth-Vázquez J. M. Navarro-Flores M. F. Martínez-Saavedra M. Juárez-León Ó. A. (2017). Fibroblast growth factor 21 and its novel association with oxidative stress. Redox Biol. 11, 335–341. 10.1016/j.redox.2016.12.024 28039838 PMC5200873

[B17] Guo T. Gupta A. Yu J. Granados J. Z. Gandhi A. Y. Evers B. M. (2021). LIFR-α-dependent adipocyte signaling in obesity limits adipose expansion contributing to fatty liver disease. iScience 24 (3), 102227. 10.1016/j.isci.2021.102227 33748712 PMC7970148

[B18] Harjumäki R. Pridgeon C. S. Ingelman-Sundberg M. (2021). CYP2E1 in alcoholic and non-alcoholic liver injury. Roles of ROS, reactive intermediates and lipid overload. Int. J. Mol. Sci. 22 (15), 8221. 10.3390/ijms22158221 34360999 PMC8348366

[B19] He Y. Feng D. Li M. Gao Y. Ramirez T. Cao H. (2017). Hepatic mitochondrial DNA/Toll-like receptor 9/MicroRNA-223 forms a negative feedback loop to limit neutrophil overactivation and acetaminophen hepatotoxicity in mice. Hepatology 66 (1), 220–234. 10.1002/hep.29153 28295449 PMC5481471

[B20] Heinke P. Rost F. Rode J. Trus P. Simonova I. Lázár E. (2022). Diploid hepatocytes drive physiological liver renewal in adult humans. Cell Syst. 13 (6), 499–507.e12. 10.1016/j.cels.2022.05.001 35649419

[B21] Huai Q. Zhu C. Zhang X. Dai H. Li X. Wang H. (2024). Mesenchymal stem/stromal cells armored by FGF21 ameliorate alcohol-induced liver injury through modulating polarization of macrophages. Hepatol. Commun. 8 (4), e0410. 10.1097/HC9.0000000000000410 38551384 PMC10984668

[B22] Huang R. Zhang X. Gracia-Sancho J. Xie W. F. (2022). Liver regeneration: cellular origin and molecular mechanisms. Liver Int. 42 (7), 1486–1495. 10.1111/liv.15174 35107210

[B23] Itoh N. Nakayama Y. Konishi M. (2016). Roles of FGFs as paracrine or endocrine signals in liver development, health, and disease. Front. Cell Dev. Biol. 4, 30. 10.3389/fcell.2016.00030 27148532 PMC4829580

[B24] Jabeen K. Malik U. Mansoor S. Shahzad S. Zahid S. Javed A. (2021). Effect of oxidative stress and calcium deregulation on FAM26F (CALHM6) expression during hepatitis B virus infection. BMC Infect. Dis. 21 (1), 228. 10.1186/s12879-021-05888-0 33639860 PMC7913464

[B25] Keinicke H. Sun G. Mentzel C. M. J. Fredholm M. John L. M. Andersen B. (2020). FGF21 regulates hepatic metabolic pathways to improve steatosis and inflammation. Endocr. Connect. 9 (8), 755–768. 10.1530/EC-20-0152 32688339 PMC7424338

[B26] Kim M. Park Y. Kim Y. S. Ko S. (2024). Cellular plasticity in gut and liver regeneration. Gut Liver 18 (6), 949–960. 10.5009/gnl240005 39081200 PMC11565004

[B27] Li J. Liang X. Jiang J. Yang L. Xin J. Shi D. (2022). PBMC transcriptomics identifies immune-metabolism disorder during the development of HBV-ACLF. Gut 71 (1), 163–175. 10.1136/gutjnl-2020-323395 33431576 PMC8666828

[B28] Li X. Li H. Zhu Y. Xu H. Tang S. (2023). PLT counts as a predictive marker after plasma exchange in patients with hepatitis B virus-related acute-on-chronic liver failure. J. Clin. Med. 12 (3), 851. 10.3390/jcm12030851 36769497 PMC9917441

[B29] Li X. Liu P. Wang Z. Wei X. Gao S. Fan Y. (2024). The value of promoter methylation of fibroblast factor 21 (FGF21) in predicting the course of chronic hepatitis B and the occurrence of oxidative stress. Virol. J. 21 (1), 332. 10.1186/s12985-024-02605-6 39710689 PMC11664819

[B30] Lima T. I. Guimarães DSPSF Oliveira A. G. Araujo H. Sponton C. H. G. Souza-Pinto N. C. (2019). Opposing action of NCoR1 and PGC-1α in mitochondrial redox homeostasis. Free Radic. Biol. Med. 143, 203–208. 10.1016/j.freeradbiomed.2019.08.006 31408725

[B31] Liu Z. Y. Luo Y. Fang A. P. Wusiman M. He T. T. Liu X. Z. (2022). High serum fibroblast growth factor 21 is associated with inferior hepatocellular carcinoma survival: a prospective cohort study. Liver Int. 42 (3), 663–673. 10.1111/liv.15100 34812573

[B32] Lu Y. Xie T. Zhang Y. Zhou F. Ruan J. Zhu W. (2017). Triptolide induces hepatotoxicity via inhibition of CYP450s in rat liver microsomes. BMC Complement. Altern. Med. 17 (1), 15. 10.1186/s12906-016-1504-3 28056947 PMC5217299

[B33] Lu Y. Li R. Zhu J. Wu Y. Li D. Dong L. (2019). Fibroblast growth factor 21 facilitates peripheral nerve regeneration through suppressing oxidative damage and autophagic cell death. J. Cell Mol. Med. 23 (1), 497–511. 10.1111/jcmm.13952 30450828 PMC6307793

[B34] Ma J. Ghabril M. Chalasani N. (2023). Drug-induced acute-on-chronic liver failure: challenges and future directions. Clin. Liver Dis. 27 (3), 631–648. 10.1016/j.cld.2023.03.007 37380287

[B35] Mansouri A. Gattolliat C. H. Asselah T. (2018). Mitochondrial dysfunction and signaling in chronic liver diseases. Gastroenterology 155 (3), 629–647. 10.1053/j.gastro.2018.06.083 30012333

[B36] Michalopoulos G. K. Bhushan B. (2021). Liver regeneration: biological and pathological mechanisms and implications. Nat. Rev. Gastroenterol. Hepatol. 18 (1), 40–55. 10.1038/s41575-020-0342-4 32764740

[B37] Ou-Yang Q. Lin X. M. Zhu Y. J. Zheng B. Li L. Yang Y. C. (2018). Distinct role of nuclear receptor corepressor 1 regulated *de novo* fatty acids synthesis in liver regeneration and hepatocarcinogenesis in mice. Hepatology 67 (3), 1071–1087. 10.1002/hep.29562 28960380 PMC6661113

[B38] Qiang W. Shen T. Noman M. Guo J. Jin Z. Lin D. (2021). Fibroblast growth factor 21 augments autophagy and reduces apoptosis in damaged liver to improve tissue regeneration in zebrafish. Front. Cell Dev. Biol. 9, 756743. 10.3389/fcell.2021.756743 34746149 PMC8570170

[B39] Qin Z. Gao L. Lin G. Zhu H. Chen Y. Zhong F. (2022). The nuclear receptor co-repressor 1 is a novel cardioprotective factor against acute myocardial ischemia-reperfusion injury. J. Mol. Cell Cardiol. 166, 50–62. 10.1016/j.yjmcc.2022.01.006 35081368

[B40] Sarin S. K. Kumar M. Lau G. K. Abbas Z. Chan H. L. Y. Chen C. J. (2016). Asian-pacific clinical practice guidelines on the management of hepatitis B: a 2015 update. Hepatol. Int. 10 (1), 1–98. 10.1007/s12072-015-9675-4 26563120 PMC4722087

[B41] Stallcup M. R. Poulard C. (2020). Gene-specific actions of transcriptional coregulators facilitate physiological plasticity: evidence for a physiological coregulator code. Trends Biochem. Sci. 45 (6), 497–510. 10.1016/j.tibs.2020.02.006 32413325 PMC7230073

[B42] Stefan N. Schick F. Birkenfeld A. L. Häring H. U. White M. F. (2023). The role of hepatokines in NAFLD. Cell Metab. 35 (2), 236–252. 10.1016/j.cmet.2023.01.006 36754018 PMC10157895

[B43] Walesky C. M. Kolb K. E. Winston C. L. Henderson J. Kruft B. Fleming I. (2020). Functional compensation precedes recovery of tissue mass following acute liver injury. Nat. Commun. 11 (1), 5785. 10.1038/s41467-020-19558-3 33214549 PMC7677389

[B44] Xie H. An C. Bai B. Luo J. Sun N. Ci B. (2025). Modeling early gastrulation in human blastoids with DNA methylation patterns of natural blastocysts. Cell Stem Cell 32 (24), 409–425.e8. 10.1016/j.stem.2024.12.010 39814012

[B45] Yang C. Lu W. Lin T. You P. Ye M. Huang Y. (2013). Activation of liver FGF21 in hepatocarcinogenesis and during hepatic stress. BMC Gastroenterol. 13, 67. 10.1186/1471-230X-13-67 23590285 PMC3637159

[B46] Yao F. Deng Y. Zhao Y. Mei Y. Zhang Y. Liu X. (2021). A targetable LIFR-NF-κB-LCN2 axis controls liver tumorigenesis and vulnerability to ferroptosis. Nat. Commun. 12 (1), 7333. 10.1038/s41467-021-27452-9 34921145 PMC8683481

[B47] Ye D. Wang Y. Li H. Jia W. Man K. Lo C. M. (2014). Fibroblast growth factor 21 protects against acetaminophen-induced hepatotoxicity by potentiating peroxisome proliferator-activated receptor coactivator protein-1α-mediated antioxidant capacity in mice. Hepatology 60 (3), 977–989. 10.1002/hep.27060 24590984

[B48] Zhang L. Wang X. Cueto R. Effi C. Zhang Y. Tan H. (2019). Biochemical basis and metabolic interplay of redox regulation. Redox Biol. 26, 101284. 10.1016/j.redox.2019.101284 31400697 PMC6831867

